# Impacts of Urbanization Undermine Nestedness of the Plant–Arbuscular Mycorrhizal Fungal Network

**DOI:** 10.3389/fmicb.2021.626671

**Published:** 2021-03-09

**Authors:** Litao Lin, Yun Chen, Guorui Xu, Yuxin Zhang, Shuang Zhang, Keming Ma

**Affiliations:** ^1^State Key Laboratory of Urban and Regional Ecology, Research Center for Eco-Environmental Sciences, Chinese Academy of Sciences, Beijing, China; ^2^University of Chinese Academy of Sciences, Beijing, China; ^3^Key Laboratory of Tropical Forest Ecology, Xishuangbanna Tropical Botanical Garden, Chinese Academy of Sciences, Mengla County, China

**Keywords:** urbanization, nestedness, network, convergent adaptation, diversity, mycorrhizal associations

## Abstract

Cities are prone to ecological problems, yet the impacts of rapid global urbanization on the feedback between above- and belowground subsystems remain largely unknown. We sampled the roots of 8 common herbaceous plants within the Fifth Ring (urban areas) and in Jiufeng National Forest Park (rural areas) in Beijing (China) to assess the impacts of urbanization on the network of plant-arbuscular mycorrhizal (AM) fungal associations. Using Illumina MiSeq sequencing, 81 AM fungal OTUs were identified in 78 herb root samples. The Shannon, Simpson, and Pielou indices of root AM fungi in urban areas were significantly higher than those in rural areas. In this study, a significantly nested mycorrhizal association network was observed in rural areas (NODF = 64.68), whereas a non-nested pattern was observed in urban areas (NODF = 55.50). The competition index C-score (0.0769) of AM fungi in urban areas was slightly lower than that in rural areas (0.1431), and the species specialization (*d*’) of 8 host plants and fungal dissimilarity among 8 host plants in urban areas were significantly lower than those in rural areas. Convergent associations among hosts may be an important factor influencing this non-nested pattern of the plant-AM fungi network in urban areas. Generalists, rather than specialists, were enhanced during the establishment of mycorrhizal associations in urban areas. Our results suggest that reduced selectivity of host plants, and generalist promotion and specialist reduction of AM fungi during urbanization may contribute to the non-nested network of plant-AM fungal associations.

## Introduction

Urban expansion is occurring worldwide (Angel et al., 2005) and may affect above- and belowground system feedback. Interactions between plants and mycorrhizal fungi are tremendously complex, and the multispecies networks resulting from these associations have consequences for plant growth and productivity (Garg and Chandel, 2010; Hacquard and Schadt, 2015). Habitat fragmentation, isolation, heavy metal pressure, high nutrient availability, and plant diversity changes that occur with rapid urbanization could present great ecological risks to plant-mycorrhizal fungal associations (Pickett et al., 2001; Jacquot-Plumey et al., 2003; Hoch et al., 2019). Currently, urban mycorrhizal studies are mainly focused on understanding the essential role of AM fungi in plant growth (Newbound et al., 2010; Chagnon and Brisson, 2017) and their diversity along urbanization gradients (Bainard et al., 2011; Martinova et al., 2016). Knowledge of how AM fungi are associated with co-occurring plant species could offer novel insights into the community-scale processes of plant–AM fungus symbioses (Chagnon et al., 2012). However, whether the network consists of host plants and their associated fungi are affected by urbanization remains largely unknown.

AM fungi assist strict associations with plants to compensate for the loss of saprotrophic capabilities (Bonfante and Genre, 2010). Associations between AM fungi and plants follow nonrandom assemblages (Montesinos-Navarro et al., 2012; Sepp et al., 2019). Roots colonized with different AM fungal species exhibit different alleviation effects on Arizona cypress seedlings under fuel pollutants (Aalipour et al., 2019). The interspecific interactions in communities with a nested network structure in which specialist species interact with subsets of species that interact with generalist species have been found to be highly robust to species extinctions (Bascompte et al., 2006; Bascompte, 2010). Significantly nested network topologies are observed in plant-AM fungal associations (Chagnon et al., 2012; Montesinos-Navarro et al., 2012). Moreover, partner selection in mycorrhizal associations could be mediated by competition, resource abundance, and microhabitat conditions (Verbruggen et al., 2012; van der Heijden et al., 2015; Lekberg and Waller, 2016) and is thus context-dependent. Experiments conducted in urban green roofs confirm that nutrient conditions can affect AM fungal association establishment (Verbruggen et al., 2012; Chaudhary et al., 2019). Some experiments have revealed no significant colonization difference between urban parks and mature forests (Karliński et al., 2014). Under similar resources, optimal foraging theory predicts selection for generalization in choosing partners (Waser et al., 1996), —the latter conflicting with the plants’ interest in specialized partners. The selective environmental filters in urbanized areas differ from those in rural areas (Knapp et al., 2008). To adapt to the urban environment, urban plants present a homogenization of the spatial plant composition (Ramalho et al., 2018). However, for mycorrhizal associations, whether urbanization raises positive or negative impacts on the nestedness of the association network remains poorly understood.

To address this research gap, we studied 8 widely distributed herbs in both urban and rural areas of Beijing (China) to investigate the impact of urbanization on the pattern of plant-AM fungal networks. In consideration of the findings of previous studies (Ramalho et al., 2018), such as the nested pattern of plant-AM fungal associations and the homogenization of the spatial plant composition in urban areas, we hypothesized that (1) the urban greenspace plant-AM fungal network possess lower levels of nestedness than the rural network and that (2) selection for generalists is the main factor accounting for the change in nestedness during urbanization. The results deepen our understanding of above- and belowground system feedback in the context of rapid global urbanization.

## Materials and Methods

### Study Site and Sampling

**FIGURE 1:**
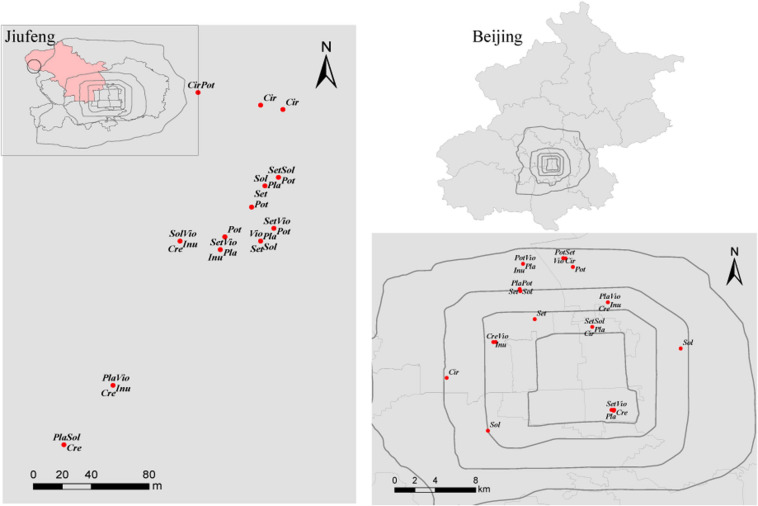
Distribution of the sampled plants in Jiufeng National Forest Park and within the Fifth Ring of Beijing. *Sol*, *Solanum nigrum*; *Pot*, *Potentilla supina*; *Cir*, *Cirsium setosum*; *Inu*, *Inula japonica*; *Pla*, *Plantago depressa*; *Cre*, *Crepidiastrum sonchifolium*; *Set*, *Setatia viridis*; *Vio*, *Viola philippica*.

This study was conducted in Beijing (39°28′-41°05′ N, 115°25′-117°30′ E), which is located in the northern part of the North China Plain. Beijing has a typical continental monsoon climate, with a mean annual precipitation of 571.8 mm and a mean annual temperature of 10–12 °C. This area has cinnamon soil with a loamy texture. Urban area is located within the Fifth Ring of Beijing. Rural area is located at Jiufeng National Forest Park (40°03′54″N, 116°05′45″E), with a forest coverage of 96.2% and approximately 30 km from the urban area of Beijing (Figure 1).

Fieldwork was performed from July to August 2013. Based on field investigation, 10 common local herbaceous species, 10 genera, 7 families (He et al., 1992), easily colonized by AM fungi were selected in urban areas. Yet, *Trigonotis peduncularis* and *Taraxacum mongolicum* failed to be sampled in the Jiufeng National Forest Park. Thus, 8 herbs *Crepidiastrum sonchifolium*, *Potentilla supina*, *Cirsium setosum*, *Setaria viridis*, *Viola philippica*, *Solanum nigrum*, *Plantago depressa*, and *Inula japonica* in rural and urban areas were used in network construction. For each herb, 5 spatially separated 20 m^∗^ 20 m plots were established in urban areas and in rural areas (Figure 1). Within each 20 m^∗^ 20 m plot, 3 1 m^∗^ 1 m subplots were established, and 3 individuals of each herbaceous species and 5 soil cores (3.5 cm diameter^∗^ 20 cm deep) were collected in each subplot. Thus, fifteen soil cores were collected from each 20 m × 20 m plot and pooled to yield a composite soil sample. The roots of the sampled plants were rinsed with tap water and then with sterilized distilled water. To reduce the workload, for each herb species, the roots of 9 plant individuals collected from a 20 m × 20 m plot were pooled to yield a composite root sample. In total, 80 root samples were collected in rural and in urban areas. All root samples were frozen and stored at −80 °C for subsequent DNA extraction.

### Soil and Plant Characteristics Analysis

The soil pH was measured at a soil/water ratio of 1:2.5 (w/v). The available phosphorus (AP) in the soils was determined using an extraction with sodium bicarbonate (Olsen, 1954). The soil organic carbon (SOC) was measured directly by dry combustion after removing the inorganic carbon by excess addition of HCl (1:1, V%). Then, soil organic carbon (SOC) and total nitrogen (TN) contents were determined by direct combustion using the Element Analyzer (Vario EL, Elementar, Germany). According to the environmental quality standard for soils and plants (GB 15618-1995), the samples was first soaked with HNO_3_-HF-HClO_4_ (6 ml+6 ml+2 ml) acid mixture for 12 h. After 180 °C by microwave digestion with an HNO_3_-HF-HclO_4_ acid mixture (Wang et al., 2013), the Mg (g⋅kg^–1^), Zn (mg⋅kg^–1^), Mn (mg⋅kg^–1^), and Cr (mg⋅kg^–1^) were determined with an ICP-OES (Optima 8300, PerkinElmer, United States); and the Cd (mg⋅kg^–1^), Ni (mg⋅kg^–1^), Cu (mg⋅kg^–1^), and Pb (mg⋅kg^–1^) were determined with an ICP-MS (7500 a, Agilent Technologies, United States).

### DNA Extraction and Sequencing

The root samples were pulverized and mixed with liquid nitrogen using a mortar that was cleaned with HNO_3_ and sterilized distilled water and sterilized in an autoclavation. Total DNA was extracted from 0.05 g of each sample using a MOBIO PowerPlant DNA extraction kit (MO Bio Laboratories, United States). The final DNA quality and contents was measured by NanoDrop (Thermo Fisher Scientific, United States). The total DNA was diluted 10 times, 20 times, and 50 times as template for the primary PCR amplifications.

The SSU regions of the nuclear ribosomal RNA genes of Glomeromycota were amplified from DNA extracts using a semi-nested PCR protocol with primer pairs AML1/AML2 (Lee et al., 2008) and NS31/AML2 that combined with adapter sequences and barcode sequences (Simon et al., 1992). Primary PCR carried out in 25 μL reaction volumes, each containing 1 μL of DNA template (10 times, 20 times, or 50 times DNA dilutions), 2.5 μL of buffer, 2 U of *Taq* DNA polymerase, 4 mM dNTPs, and each primer at 4 μÌ. The following cycling parameters were used: 94°C for 5 min; 35 cycles at 94°C for 30 s, 58°C for 45 s, and 72°C for 1 min; and a final step at 72°C for 10 min. PCR products of 3 replications were mixed and diluted 10 times with double-distilled H_2_O as the DNA template for the second PCR amplifications. The second PCR carried out in 50 μL reaction volumes, each containing 5 μL of DNA template, 5 μL of 10 × buffer, 2 U of *Taq* DNA polymerase, 10 mM dNTPs, each primer at 8 μÌ, and double-distilled H_2_O to a final reaction volume of 50 μL. The second PCR was performed under the conditions as the first one except that 30 cycles was used. The PCR products were examined on ethidium bromide-stained 2% agarose gels by electrophoresis and visualized under UV light. To minimize heterogeneity, three replicates of the second PCR products from each sample were pooled together. This DNA mix was purified with a AxyPrepDNA Gel Extraction Kit (AXYGEN, United States) and equal concentrated amplicons were sequenced on an Illumina MiSeq PE 300 platform (Illumina, San Diego, CA, United States).

### Bioinformatics Analyses

The sequence reads were processed with a QIIME toolkit (Caporaso et al., 2010). First, sequence reads were quality-filtered and demultiplexed according to the following criteria: minimum length ≥ 200 bp (excluding barcode and primer sequences); ambiguous bases ≤ 0; homopolymer length ≤ 10 bp; maximum number of primer or barcode mismatches ≤ 0; and minimum mean quality score ≥ 30 in a window of 50 nt. The 80 root samples yielded a total of 1 757 618 sequences after quality control, with an average sequence length of 291 bp. Using the *de novo* approach, we detected 9 232 chimeric sequences, and the remaining non-chimeric sequences yielded 164 initial OTUs based on 97% sequence similarity using the USEARCH algorithm. The OTUs sequences were then subjected to a BLASTN search of the SILVA database (Pruesse et al., 2007). Through comparison using the SILVA database, 1 323 639 sequences were identified as Glomeromycota. However, the pot-U1 and pot-R4 samples only yielded 3 and 7 Glomeromycota sequences and were excluded from the subsequent processing (Supplementary Table S1). Clusters representing < 0.0001% of the total reads were removed in subsequent analysis to avoid α diversity overestimation due to sequencing errors (Bokulich and Mills, 2013). For the comparative analyses, the OTU table was rarefied without replacement to 4 227 reads (the lowest number of sequences among all the samples) per sample. Multiple sequence alignment and construction of the maximum likehood (ML) phylogenetic tree were performed using MEGA6.0 (Tamura et al., 2013). The robustness of this phylogenetic tree was evaluated using 1 000 bootstrap replications. The raw reads have been submitted to the NCBI (National Center for Biotechnology Information) Sequence Read Archive (SRA) database under accession numbers PRJNA64452.

### Statistical Analyses

Using the vegan package (Oksanen et al., 2019) of R software (https://cran.r-project.org), we calculated Shannon’s diversity {*H* = −∑*p*_i_[ln ⁡(*p*_i_)]}, Simpson’s diversity ($D=1-\sum p_{i}^{2}$), Pielou’s diversity (*E* = *H*/ ln (*S*)). We used the picante package (Kembel et al., 2010) to calculate Faith’s PD phylogenetic diversity (PD), where *S* is the number of OTUs in each sample and *p*_*i*_ representes a single OTU sequence abundance ratio of each sample’s total abundance. A linear mixed effects model (LMM) in the nlme package (Pinheiro et al., 2018) was used to measure differences in plant heavy metal contents, plant individual biomass, relative abundance of fungal OTUs, and fungal alpha diversity between the urban and rural samples, with the land use type (urban/rural) set as a fixed effect and plant species set as a random effect. Difference of soil properties in rural areas and those in urban areas were tested by one-way ANOVA.

The impacts of urbanization on AM fungal community structure were measured by Adonis (permutational multivariate analysis of variance), ANOSIM (analysis of similarity), and MRPP (multiresponse permutation procedure) tests via the vegan package, with 999 random permutations. The homogeneity of the variances were checked using the function betadisper in vegan package. Fungal dissimilarity among 8 host species were measure by Bray-Curtis distance (*BC*). $B\,C=\frac{\sum_{\text{k}=1}^{\text{n}}\left|x_{i\,k}-x_{j\,k}\right|}{\sum_{k=1}^{n}\left(x_{i\,k}+x_{j\,k}\right)}$ where *i* and *j* represented different host species, x was the sequence number of the *k*-th OTU, and *n* was the sum of the total number of OTUs in all the samples. Difference between the fungal dissimilariy (*BC*) among host plants in rural areas and that in urban areas was tested by the paired sample t test.

The original observed data consisted of a matrix with herbaceous plants in rows and AM fungi being aggregated within each plant species in columns. Therefore, the network in this study represents plant-symbiotic fungal associations (Chagnon et al., 2012; Toju et al., 2016). The NODF (nestedness metric based on paired overlap and decreasing fill) was used to measure the nestedness of mycorrhizal interactions (Almeida-Neto et al., 2008). $N\,O\,D\,F=\frac{\sum N_{p\,a\,i\,r\,e\,d}}{\left[\frac{n\,\left(n-1\right)}{2}\right]\,\left[\frac{m\,\left(m-1\right)}{2}\right]}$, where *m* is number of rows; *n* is number of columns; row *i* is located at an upper position from row *j*; and column *k* is located at a left position from column *l*. $N_{paired}=\{{}_{PO,\;if\;DF_{paired}=100}^{\;0,\;if\;DF_{paired}=0}$, where marginal total of row *j* (column *l*) less than marginal total of row *i* (column *k*) *DF*_*paired*_ = 0; marginal total of row *j* (column *l*) no less than marginal total of row *i* (column *k*) *DF*_*paired*_ = 100; *PO* is percentage of co-present 1’s in column (row) pairs to those in a column *k* (row *i*). In this study, all AM fungi in rural or urban areas were considered during nesting calculation, for no significant alpha and beta diversity difference being observed between soil AM fugnal pools (Lin et al., 2020). NODF calculations and significances tests using 50 replicates of column-random row (EE) nulls and 50 replicates of fixed column-fixed row (FF) nulls were conduceted in ANINHADO software (Guimarães and Guimarães, 2006). EE null: presences are randomly assigned to any cell within the matrix. FF null: the probability of a cell *a*_*ij*_ show a presence is $\left(\frac{P_{i}}{C}\,\frac{P_{j}}{R}\right)/2$, in which *P*_*i*_ is the number of presences in the row *i*; *P*_*j*_ is the number of presences in the column *j*; *C* is the number of columns; *R* is the number of rows. The NODF_c is propsed to compare nestedness level betweeInclude the following n netoworks by Song et al. (2017). *NODF*_*c* = *NODF*/*max*(*NODF*)/(C*log(*S*)), where *max(NODF)* is the maximum nestedness in a network with given rows, columns and links; *C* is the network connectance; *S* is the geometric mean number of species in the network. NODFc of observed network and significances tests using 999 replicates of EE nulls were measured via maxnodf package (Christoph and Benno, 2020) and vegan package.

Using bipartite package (Dormann et al., 2018), we calculate the *degree*, *d*’, *H*_2_’, and *C.score* metrics to describe the network topology based on the mycorrhizal interaction matrices. *Degree* is the number of links per species, and values range from 1 (specialist) to the number of hosts (generalist) (Gonzalez et al., 2010). The significances of plant-AM fungal links in networks and *d*’ of AM fungi were tested by using 999 replicates of swap.web null in bipartite package. Standardized effect size (*z-score*), $\frac{o\,b\,s-m\,e\,a\,n\,\left(n\,u\,l\,l\,s\right)}{s\,d\,\left(n\,u\,l\,l\,s\right)}$, where *obs* is the observed links (or *d*’ value) of a fungus; *mean(nulls)* is the mean value of the links (or *d*’ value) from 999 swap.web randomizations; *sd(nulls)* is the standard deviation of the links (or *d*’ value) from randomizations. *Z-score* > 1.96 (<-1.96) indicate that the observed links or *d*’ value of a fungus is 0.05 significantly higher (lower) than that of randomizations.

Species specialization *(d*’) & overall degree of specialization (*H*_2_’) are quantitative indices used to describe the degree of interaction specialization,: *d*’ characterizes specialization at the species level, while *H*_2_’ characterizes specialization at the network level (Blüthgen et al., 2006). The *d*’ index emphasizes the intensity of a species deviating from a random sampling, calculated as the Kullback-Leibler distance. Values range from 0 (generalist) to 1 (specialist). *D*’ is calculated first by finding *d*_*i*_: $d_{i}=\sum_{j=1}^{c}p_{i\,j}^{\prime }\,l\,n\,\frac{p_{i\,j}^{\prime }}{q_{j}}$, where *c* is the range of resources; *p*’_*ij*_ is the interactions of species *i*/ the sum of performances of species *i*; *q*_*j*_ is the sum of interactions of resource *j*/ the total number of interactions in the matrix. Then, $d^{\prime }=\frac{d_{i}-d_{m\,i\,n}}{d_{m\,a\,x}-d_{m\,i\,n}}$, where *d*_*max*_ is given analytically, and *d*_*min*_ is found heuristically. *H*_2_’ is the extension of *d*’ for the entire association network. In this study, *H*_2_’ calculates how strongly the observed interactions deviating from random expectation with the given species’ marginal totals. *C.score* calculates the (normalised) mean number of checkerboard combinations. Values range from 0 (aggregation, no repelling forces between species) to 1 (evidence for disaggregation, e.g. through competition).

## Results

### Characterization of Soil Properties

Soil properties were significantly different between rural and urban sites (Table 1). The pH, total nitrogen (TN), magnesium (Mg), nickel (Ni), copper (Cu), and lead (Pb) contents in urban soil were significantly higher than those in rural soil (*P* < 0.05). No significant differences between soil organic carbon (SOC), available phosphorus (AP), chromium (Cr), cadmium (Cd), zinc (Zn), and manganese (Mn) contents were observed between urban sites and rural sites. Herbaceous richness in urban areas was significantly lower than that in rural areas (*P* < 0.05).

**TABLE 1:** Mean (SE) values of soil properties and herbaceous richness in rural and urban areas.

Area	n	pH	SOC (%)	TN (g⋅kg^–1^)	AP (mg⋅kg^–1^)	Mg (g⋅kg^–1^)	Cr (mg⋅kg^–1^)
Rural	13	8.20 (0.15)a	0.10 (0.003)	1.04 (0.04)a	11.44 (2.07)	8.33 (0.31)a	70.70 (5.93)
Urban	30	8.63 (0.02)b	0.09 (0.007)	2.10 (0.08)b	16.24 (2.92)	9.67 (0.21)b	84.33 (6.21)
	Cd (mg⋅kg^–1^)	Ni (mg⋅kg^–1^)	Zn (mg⋅kg^–1^)	Mn (mg⋅kg^–1^)	Cu (mg⋅kg^–1^)	Pb (mg⋅kg^–1^)	Herbaceous richness
Rural	0.98 (0.01)	21.17 (0.67)a	60.22 (3.77)	446.62 (17.28)	17.65 (0.54)a	19.17 (0.44)a	62.66 (2.76)b
Urban	1.07 (0.06)	26.79 (0.96)b	62.33 (4.40)	451.29 (9.49)	23.49 (1.37)b	23.90 (1.08)b	20.64 (1.64)a

*n, the number of sampled 20 m × 20 m plots; SOC, soil organic carbon; TN, total nitrogen; AP, available phosphorus; Mg, magnesium; Cr, chromium; Cd, cadmium; Ni, nickel; Zn, zinc; Mn, manganese; Cu, copper; Pb, lead. Letters within column indicate 0.05 significant differences.*

**FIGURE 2:**
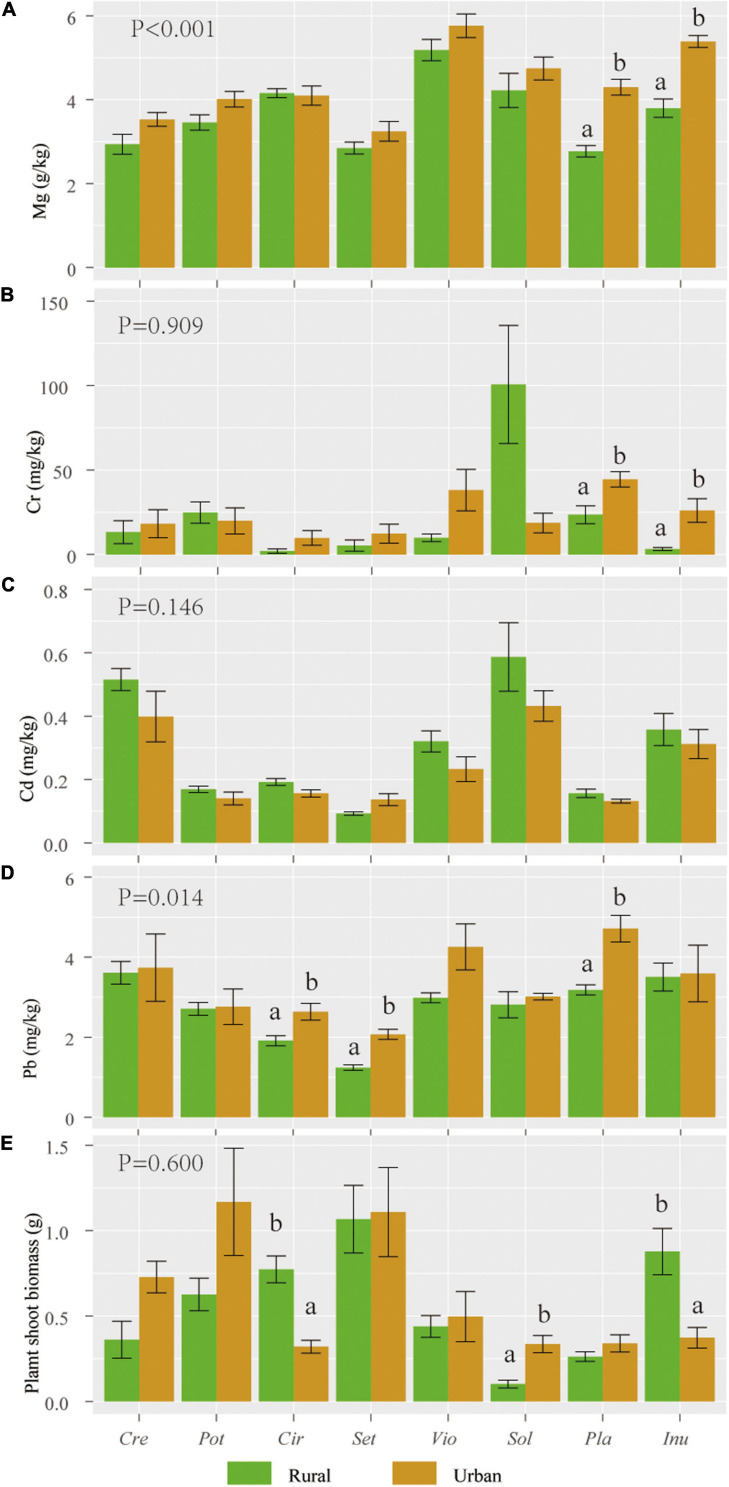
Mean (standard error, SE) values of Mg **(A)**, Cr **(B)**, Cd **(C)**, and Pb **(D)** contents and individual biomass **(E)** of 8 host species in rural and urban areas (*n* = 5). Abbreviations in the X axis: *Sol*, *Solanum nigrum*; *Pot*, *Potentilla supina*; *Cir*, *Cirsium setosum*; *Inu*, *Inula japonica*; *Pla*, *Plantago depressa*; *Cre*, *Crepidiastrum sonchifolium*; *Set*, *Setatia viridis*; *Vio*, *Viola philippica*. Different letters indicate 0.05 LSD significant differences. *P* values indicate LSD differences in heavy metal content and biomass of plant species in urban or rural areas (*n* = 8).

The magnesium (Mg) and lead (Pb) contents of urban plants were significantly higher than those of rural plants (*P* < 0.05). The individual biomass, chromium (Cr), and cadmium (Cd) levels of urban plants were not significantly different from those of rural plants (Figure 2).

### Characterization of Fungal Diversity During Urbanization

**FIGURE 3:**
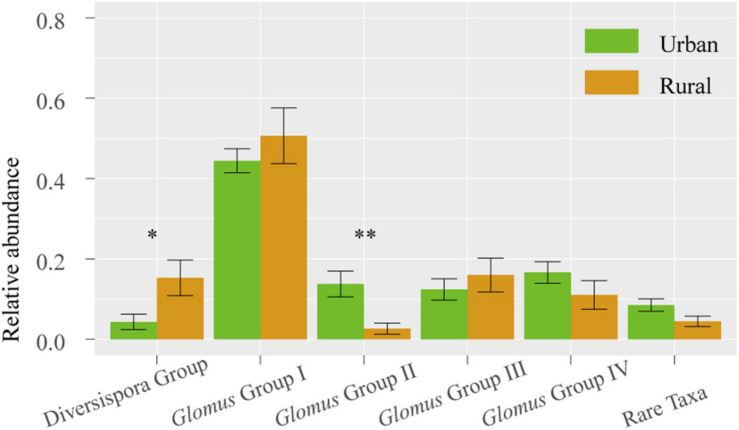
Relative abundances of AM fungal groups in urban and rural samples. The vertical bars represent the standard error of the mean (*n* = 8). *LSD significant at the 0.05 level; **LSD significant at the 0.01 level.

Across 78 plant samples, 81 AM fungal OTUs (1 323 639 sequences) were identified. Among these OTUs, *Glomus* (65 OTUs) was the most common genus, followed by the genera *Claroideoglomus* (5 OTUs), *Gigaspora* (1 OTU), *Archaeospora* (1 OTU), *Ambispora* (1 OTU), and *Paraglomus* (1 OTU). The 81 OTUs were divided into 6 phylogenetic groups: *Glomus* Group I, *Glomus* Group II, *Glomus* Group III, *Glomus* Group IV, *Diversispora* Group, and Rare Taxa (Supplementary Figure 1). The relative abundance of *Glomus* Group II was significantly higher in urban samples than in rural samples (*P* < 0.01), whereas the *Diversispora* Group showed significantly lower relative abundance in urban areas (*P* < 0.05) (Figure 3).

**FIGURE 4:**
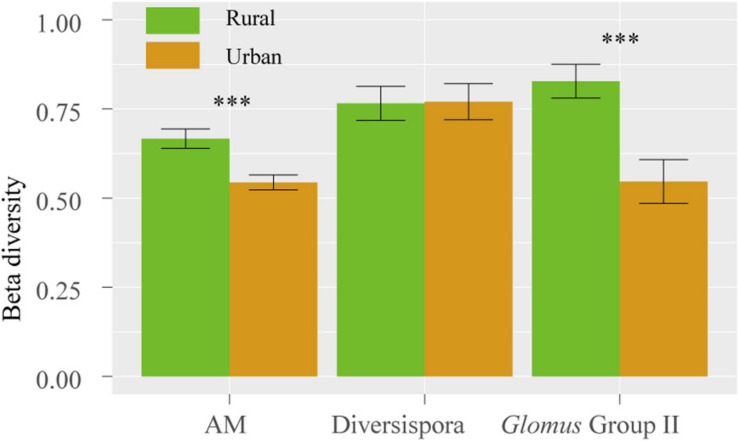
Dissimilarity of the fungal community among 8 host species in rural and urban areas. The vertical bars represent the standard error of the mean (*n* = 28). ***, LSD significant at the 0.001 level.

The Shannon, Simpson, and Pielou indices of the root AM fungi in the urban areas were significantly higher than those in the rural areas (Table 2). The communities of urban samples were significantly different from those of rural samples (Table 3). Dissimilarities in the fungal community and *Glomus* Group II among 8 host plants in urban areas showed significantly lower values than those in rural areas (Figure 4).

**TABLE 2:** LMM results the responses of AM fungal α diversity indices to land use.

Response variables (Y)	Model = lme (Y∼ Land use, random = ∼1| Species)				
	Intercept	Slope (Rural-Urban)	Degree of freedom (df)	*t*-value	*P*-value
Shannon’s diversity	1.52	0.31	69	2.22	0.0296*
Simpson’s diversity	0.46	0.11	69	2.25	0.0275*
Faith’s PD	0.44	−0.01	69	−0.09	0.9280
Pielou’s evenness	0.32	0.07	69	2.06	0.0436*

Use data of 78 plant samples. *Significant at the 0.05 level.

**TABLE 3:** Multivariate analysis of beta diversity based on the taxonomic metric and the phylogenetic metric.

Distance metric	Plant species	Adonis		ANOSIM		MRPP	
		R^2^	*P* values	R	*P* values	A	*P* values
Bray-Curtis	*Cre*	0.19	0.0050**	0.29	0.0140*	0.05	0.0210*
	*Set*	0.08	0.7920	–0.09	0.7860	–0.02	0.7410
	*Vio*	0.17	0.0990	0.20	0.0610	0.04	0.1670
	*Inu*	0.13	0.2440	0.06	0.2540	0.01	0.2370
	*Pot*	0.29	0.0600	0.24	0.0860	0.17	0.0630
	*Sol*	0.11	0.5130	0.02	0.3510	–0.00	0.5650
	*Cir*	0.08	0.8630	–0.10	0.8150	–0.02	0.9110
	*Pla*	0.18	0.1080	0.17	0.1420	0.04	0.1000
	All samples	0.04	0.0010**	0.08	0.0010**	0.01	0.0010**
Unweighted UniFrac	*Cre*	0.15	0.0860	0.16	0.0870	0.03	0.0870
	*Set*	0.11	0.5270	–0.02	0.5430	0.00	0.4020
	*Vio*	0.13	0.2250	0.108	0.2080	0.02	0.1950
	*Inu*	0.11	0.4150	–0.00	0.4360	–0.01	0.5370
	*Pot*	0.33	0.0260*	0.68	0.0360*	0.13	0.0170*
	*Sol*	0.09	0.5800	–0.11	0.7110	–0.02	0.6370
	*Cir*	0.08	0.7390	–0.09	0.7290	–0.01	0.7320
	*Pla*	0.11	0.4420	0.04	0.2960	–0.01	0.6900
	All samples	0.00	0.9420	–0.03	0.9630	–0.01	0.9570
Weighted UniFrac	*Cre*	0.23	0.0120*	0.24	0.0150*	0.07	0.0090**
	*Set*	0.06	0.8610	–0.08	0.6540	–0.04	0.7950
	*Vio*	0.16	0.1750	0.12	0.1900	0.04	0.1890
	*Inu*	0.11	0.4010	–0.04	0.5200	–0.01	0.5010
	*Pot*	0.23	0.0580	–0.00	0.5450	0.10	0.0500
	*Sol*	0.08	0.7090	–0.05	0.6480	–0.03	0.7480
	*Cir*	0.08	0.7300	–0.05	0.6300	–0.03	0.7430
	*Pla*	0.18	0.0470*	0.07	0.2530	0.04	0.0970
	All samples	0.02	0.2720	0.03	0.0340	0.01	0.0600

Use data of 78 plant samples. *Cre*, *Crepidiastrum sonchifolium*; *Set*, *Setatia viridis*; *Vio*, *Viola philippica*; *Inu*, *Inula japonica*; *Pot*, *Potentilla supina*; *Sol*, *Solanum nigrum*; *Cir*, *Cirsium setosum*; *Pla*, *Plantago depressa*. Use data of 78 plant samples. *significant at the 0.05 level; **Significant at the 0.01 level.

### Impact of Urbanization on Network Structure

**FIGURE 5:**
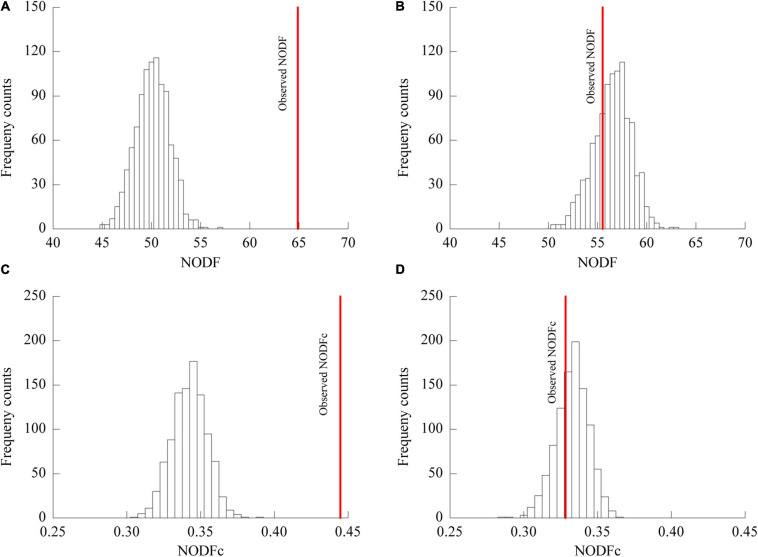
NODF and NODFc values of the network of plant-AM fungal associations in rural **(A,C)** and urban areas **(B,D)**. Bars show the distributions of mean NODF **(A,B)** and NODFc **(C,D)** values resulting from 999 EE randomizations of the respective rural **(A,C)** and urban **(B,D)** data matrices; the solid line indicates the observed NODF values in rural **(A)** and urban **(B)** areas and NODFc values in rural **(C)** and urban **(D)** areas. In **(A,C)**, the observed NODF and NODFc were higher than expected from randomization.

The plant-AM fungal network in the urban area showed a higher connectance (0.5895) level and lower H2’ (0.3972) and C.score (0.1431) values than those in the rural area (connectance = 0.5093, H2’ = 0.2619, C.score = 0.0769) (Table 4). The association network in urban areas showed a significantly lower NODF value (55.50) than that in rural areas (64.68) (Figure 5). The NODF of the rural network was significantly higher than those of the EE nulls and FF nulls (*P* < 0.05), while the urban network exhibited a non-nested pattern (*P* > 0.05) (Table 4). The NODFc value of the urban network (0.3284) was lower than that of the rural network (0.4446) and did not differ from that of the simulated EE nulls (*P* > 0.05) (Figure 5).

**TABLE 4:** Descriptive statistics of the plant–AM fungal networks in rural and urban areas.

Network	Connectance	C.score	H2’	NODF	Null_EE_	*P*_EE_	Null_FF_	*P*_FF_
Urban	0.5895	0.0769	0.2619	55.50	57.05	0.72	63.67	1.00
Rural	0.5093	0.1431	0.3972	64.68	50.44	0.00	57.89	0.02

**FIGURE 6:**
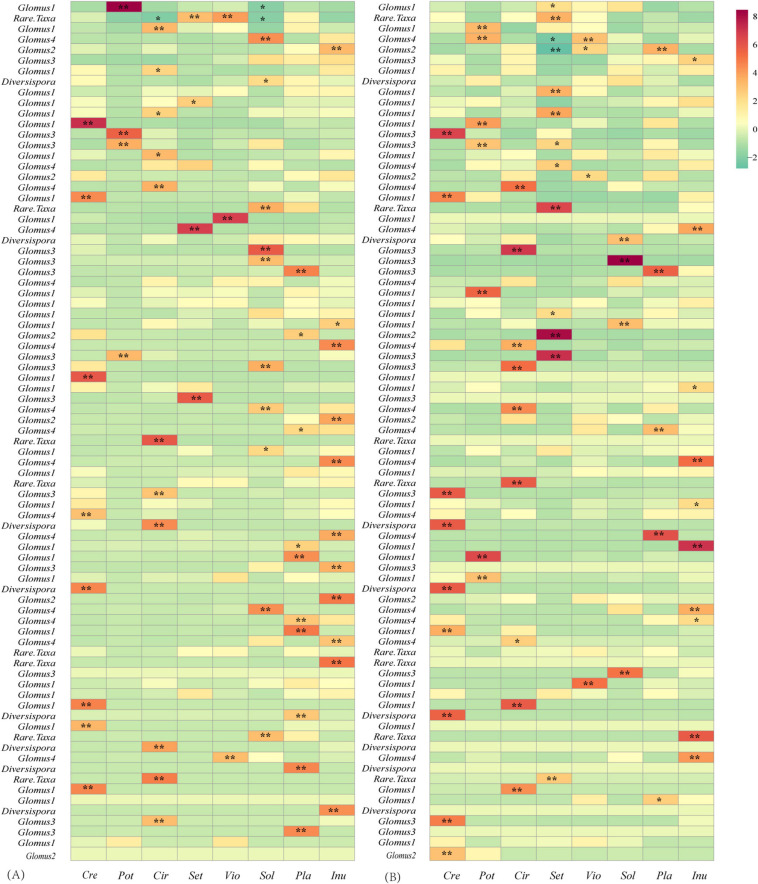
Z-score values of the links between plants and AM fungi in the rural **(A)** and urban **(B)** networks. Values > 1.96, 0.05 significantly higher than randomizations; 1.96 > values > -1.96, neutral to randomizations; values < -1.96, 0.05 significantly lower than randomizations. *, significant at the 0.05 level; **, significant at the 0.01 level.

**FIGURE 7:**
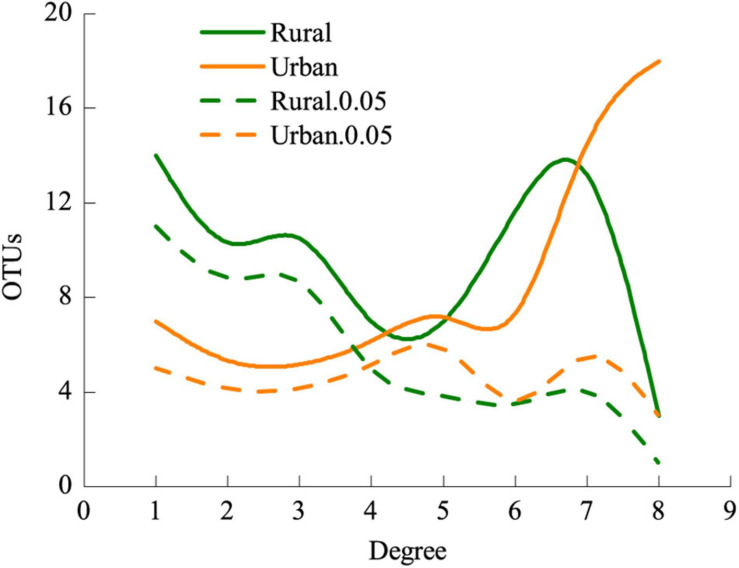
Frequencies of AM fungi and significantly specialized AM fungi of different *degrees* in rural and urban networks. Values of *degree* range from 1 (specialist) to 8 (generalist). Rural.0.05, the *d*’ of AM fungi in rural areas 0.05 significantly deviated from that of randomizations; Urban.0.05, the *d*’ of AM fungi in urban areas 0.05 significantly deviated from that of randomizations.

The LMM analysis showed that the *d*’ values of the rural plants were significantly higher than those of the urban plants (*P* < 0.05) (Table 5). The pattern of significant plant-AM fungal links differed between the rural and urban networks (Figure 6). Urbanization enhanced the percentage of generalist OTUs (*degree* = 8) and reduced the proportion of specialist OTUs (*degree* = 1) (Figure 7).

**TABLE 5:** Species specialization (*d*’) values of each plant species.

Network	*Cre*	*Pot*	*Cir*	*Set*	*Vio*	*Sol*	*Pla*	*Inu*
Urban	0.2541	0.2730	0.2318	0.4906	0.1864	0.2627	0.2044	0.1925
Rural	**0.3593**	**0.6521**	**0.3726**	0.3961	**0.3390**	**0.4655**	0.2034	**0.2418**

**Cre*, *Crepidiastrum sonchifolium*; *Set*, *Setatia viridis*; *Vio*, *Viola philippica*; *Inu*, *Inula japonica*; *Pot*, *Potentilla supina*; *Sol*, *Solanum nigrum*; *Cir*, *Cirsium setosum*; *Pla*, *Plantago depressa*. Rural plant species with a bigger *d*’ value than urban is emphasized in bold.*

## Discussion

### The Nestedness of Mycorrhizal Networks Differs Between Rural and Urban Areas

In the rural areas, the network of mycorrhizal associations showed a significantly nested pattern (Figures 4, 5). This asymmetric interactive pattern could contribute to the maintenance of biodiversity (Bascompte et al., 2006; Jabot and Bascompte, 2012). In a previous study, a 10-plant–47-AM fungi network in a hemiboreal forest also showed a significantly nested web structure (Chagnon et al., 2012). However, experiments carried out by Toju et al. (2014) suggest a non-nested 33-plant–10-AM fungal network in a temperate secondary forest. This non-nested pattern could be attributed to using non-AM specific primers and nonherbaceous hosts and only 14 out of 33 host plants were found to form associations with AM fungi. Thus, fewer co-shared fungal OTUs and lower network nestedness might be observed. Some meta-community analyses suggested that soil nutrient conditions could be a driving force of nestedness (Verbruggen et al., 2012). Habitat heterogeneity could result in nonrandom species spatial distribution in pollination networks (Olesen et al., 2007). In this study, plant roots were sampled in a large geographic and coincidental area. No significant correlation was observed between the rank of nestedness of plants and beta diversity of AM fungi (Supplementary Table S2). Thus, the effect of sampling heterogeneity was not the essential factor contributing to this nested pattern of rural networks.

In contrast to the observation that network structure shows some resistance to fluctuations in species and interactions (Petanidou et al., 2008; Montesinos-Navarro et al., 2012), urbanization disrupted the significantly nested pattern of the plant-AM fungal network (Figure 5, Table 4). NODF exhibits positive correlations with network connectance (Almeida-Neto et al., 2008). Bascompte et al. (2003) suggested that an increased number of interactions within a given number of species yields a higher relative nestedness level. However, a similar pattern was only observed in the NODF_*EE*_ nulls, and the rural network with a lower connectance level showed a higher NODF value than that of the urban network (Table 4). Thus, a large relative nestedness difference existed between the urban and rural networks. The connectance and network size independent metric NODFc also indicated that a less nested pattern was observed in urban areas (0.3284) than in rural areas (0.4446).

In this study, the significantly increased *d*’ of host plants (Table 5) and significantly lower fungal beta diversity among 8 host plants in the urban areas (Figure 4) than those in the rural areas, indicate a reduced selectivity of urban plants in choosing partners. It is hypothesized that the numbers of nondominant microbial species decline as soil incompatibility increases (Encinas-Viso et al., 2016). In this study, significantly higher soil Pb, Ni and Cu and plant Pb contents were observed in urban areas than those in rural soils and plants (Figure 2, Table 1). The toxicity of soil heavy metals may hinder the ranges of partner selection. Due to similar adaptations to the urban environment, urban plants show a homogenization of the spatial community composition (Knapp et al., 2008; Ramalho et al., 2018), and a similar convergent pattern was also observed in root AM fungal associations.

The frequency of generalist fungal OTUs but not that of specialist fungal OTUs was slightly enhanced in the urban areas (Figure 7). AM fungi do not strictly host specific taxa (Sanders, 2003), and habitat fragmentation during urbanization could obstruct the diffusion and colonization of specialists (Telford et al., 2006). Carbon resource or root niche heterogeneity catalyzes expansion in resource use (niche expansion) and diversification of specialist AM fungi (Dickie, 2007; Yu et al., 2018). Filters during urbanization, such as heavy metal stresses, particle deposition, and habitat fragmentation, contribute to the reduction of plant richness (Table 1) and homogenization of plant communities (Knapp et al., 2008; Ramalho et al., 2018), thus hindering the plants from associating with specialist AM fungi. Nonetheless, if specialist fungi constitute a small fraction of the overall fungal richness or are rare, nestedness is difficult to detect (Bahram et al., 2014). All these processes may explain the reduction in nested interaction patterns at the network level (Figure 5, Table 3).

### Changes in the Strength of Mycorrhizal Associations Under Urbanization

Significantly higher Shannon’s diversity and Simpson’s diversity of root AM fungi were observed in urban areas than in rural areas (Table 2). Many studies have reported the significant role of AM fungi in improving host plant resistance to heavy metal toxicity and the disease and insect resistance of host plants (Hildebrandt et al., 2007; Khade and Adholeya, 2008; Joy, 2013; Schneider et al., 2016). In this study, significantly higher soil nutrient availability and soil Mg, Ni, Cu, and Pb contents were observed in urban areas (Table 1). Plants are expected to be more dependent on mycorrhizal symbioses in urban areas than in rural areas due to the frequent exposure of plants to biotic and abiotic stresses in urban areas (Pourrut et al., 2011; Alzetta et al., 2012). Thus, the trade-off strategy (Lipson et al., 2009) supports that plants with similar biomass in urban areas yield a higher diversity of AM fungal communities under environmental pressures than those in rural areas (Table 1, Table 2).

In this study, lower demands of nutrients and higher demands of environmental stress tolerance in urbanized areas suggest a significantly different fungal composition (Table 3). The relative abundance of *Glomus* Group II was significantly enhanced during urbanization (Figure 3); this group often appears in degraded ecosystems and has a strong adaptability to environmental changes (Supplementary Table S3). Furthermore, the beta diversity of *Glomus* Group II and AM fungi among the 8 host species in urban areas was significantly lower than that in rural areas (Figure 4). Therefore, selective filters of environmental stress resistance or tolerance may play an important role in the establishment of root mycorrhizal associations (Knapp et al., 2008). The patterns of significant plant-AM fungal links in the networks were quite different between the rural and urban areas (Figure 6). Competition is a driving force of niche differentiation (Wright, 2002). In this study, urbanization reduced the level of competition among AM fungi, with a lower C-score value (0.0769) obtained for the urban network than for the rural network (0.1431) (Table 4). Fewer specialist OTUs and more generalist OTUs were observed in the urban areas (Figure 7). Thus, plants in urban areas may tend to cooperate with many kinds of available, robust AM fungi to reduce the risk of species loss (Table 1 and Figures 5, 7), other than diffusion and selectivity among host species.

## Conclusion

The rural mycorrhizal association network shows a significantly nested network structure and is highly robust to species extinctions. Heavy metal stress, habitat fragmentation, and host diversity changes during urbanization may hinder fungal specialists and enhance fungal generalists in the network of mycorrhizal associations. Urban plants may tend to cooperate with many kinds of generalist AM fungi to compensate the risks of diffusion isolation and species extinctions. All these reductions in selectivity contribute to the non-nested plant–AM fungal network in urban areas.

## Data Availability Statement

The datasets presented in this study can be found in online repositories. The names of the repository/repositories and accession number(s) can be found below: https://www.ncbi.nlm.nih.gov/, PRJNA64452.

## Author Contributions

LL performed methodology, formal analysis, writing original draft, and writing review and editing. YC performed methodology, investigation, and formal analysis. GX performed methodology. YZ performed methodology. SZ performed methodology. KM performed methodology and writing review and editing. All authors contributed to the article and approved the submitted version.

## Conflict of Interest

The authors declare that the research was conducted in the absence of any commercial or financial relationships that could be construed as a potential conflict of interest.

## Supplementary Material

Supplementary Figure 1 Maximum likelihood phylogenetic tree of the 81 AM fungal operational taxonomic units (OTUs) found in 78 root samples taken from rural and urban areas.

Supplementary Table 1 Properties of 80 plant samples in this study.

Supplementary Table 2 Linear regression of the plant rank of nestedness to sampling spatial distance, environmental distance, and fungal variation in urban and rural areas.

Supplementary Table 3 Description of sequences corresponding to the closest matches from the reference database of the representative sequence of each OTU in *Glomus* Group II.
