# Supplementary figures and images for: Impacts of Urbanization Undermine Nestedness of the Plant–Arbuscular Mycorrhizal Fungal Network
Source: Front Microbiol. 2021 Mar 9;12:626671. doi: 10.3389/fmicb.2021.626671 (PMC7985257; doi:10.3389/fmicb.2021.626671)

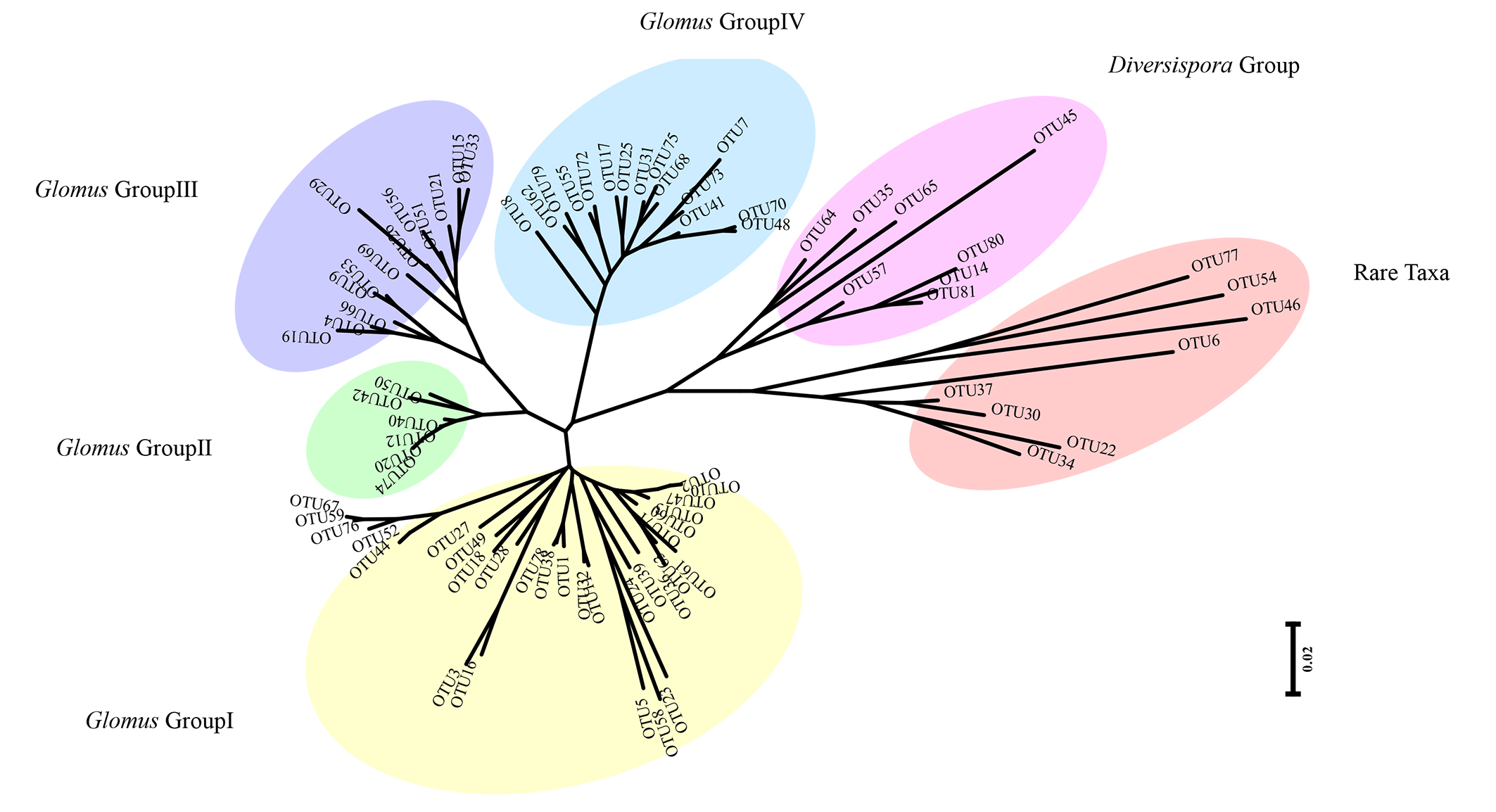

Supplement: Supplementary Figure 1 — Maximum likelihood phylogenetic tree of the 81 AM fungal operational taxonomic units (OTUs) found in 78 root samples taken from rural and urban areas. [file Image_1.jpeg]
