# Supplementary material for: Impacts of Urbanization Undermine Nestedness of the Plant–Arbuscular Mycorrhizal Fungal Network
Source: Front Microbiol. 2021 Mar 9;12:626671. doi: 10.3389/fmicb.2021.626671 (PMC7985257; doi:10.3389/fmicb.2021.626671)
Supplement: Supplementary Table 1 — Properties of 80 plant samples in this study. [file Data_Sheet_1.docx]

**Supplemental Table 1 Properties of 80 plant samples in this study**

| Sample | Land use | Latitude, longitude | | Locality | Phenological period |
| --- | --- | --- | --- | --- | --- |
| cre-U1 | Urban | 40°00′28.0″ N | 116°20′14.0″ E | RCEES, Chinese Academy of Sciences | Reproductive |
| cre-U2 | Urban | 40°00′45.0″ N | 116°22′30.0″ E | Olympic Forest Park | Reproductive |
| cre-U3 | Urban | 39°58′27.0″ N | 116°24′45.0″ E | East Yinghuayuan Street | Reproductive |
| cre-U4 | Urban | 39°56′22.0″ N | 116°18′47.0″ E | Black Bamboo Park | Reproductive |
| cre-U5 | Urban | 39°52′49.0″ N | 116°25′05.0″ E | Longtan Road | Reproductive |
| set-U1 | Urban | 40°00′45.8″ N | 116°22′22.3″ E | Olympic Forest Park | Vegetative |
| set-U2 | Urban | 39°59′02.1″ N | 116°20′05.4″ E | Beihang University | Reproductive |
| set-U3 | Urban | 39°52′49.0″ N | 116°25′05.0″ E | Longtan Road | Reproductive |
| set-U4 | Urban | 39°57′09.6″ N | 116°23′55.1″ E | Qingnianhu Park | Reproductive |
| set-U5 | Urban | 39°57′34.0″ N | 116°20′51.3″ E | Jimenqiao | Reproductive |
| vio-U1 | Urban | 40°00′28.0″ N | 116°20′14.0″ E | RCEES, Chinese Academy of Sciences | Reproductive |
| vio-U2 | Urban | 40°00′45.0″ N | 116°22′30.0″ E | Olympic Forest Park | Reproductive |
| vio-U3 | Urban | 39°58′27.0″ N | 116°24′45.0″ E | East Yinghuayuan Street | Reproductive |
| vio-U4 | Urban | 39°56′22.0″ N | 116°18′47.0″ E | Black Bamboo Park | Reproductive |
| vio-U5 | Urban | 39°52′49.0″ N | 116°25′05.0″ E | Longtan Road | Reproductive |
| inu-U1 | Urban | 40°00′28.0″ N | 116°20′14.0″ E | RCEES, Chinese Academy of Sciences | Vegetative |
| inu-U2 | Urban | 40°00′45.0″ N | 116°22′30.0″ E | Olympic Forest Park | Vegetative |
| inu-U3 | Urban | 39°58′27.0″ N | 116°24′45.0″ E | East Yinghuayuan Street | Vegetative |
| inu-U4 | Urban | 39°56′22.0″ N | 116°18′39.0″ E | Black Bamboo Park | Vegetative |
| inu-U5 | Urban | 39°52′48.6″ N | 116°24′56.8″ E | Longtan Road | Vegetative |
| pot-U1 | Urban | 40°00′28.0″ N | 116°20′14.0″ E | RCEES, Chinese Academy of Sciences | Reproductive |
| pot-U2 | Urban | 40°00′45.8″ N | 116°22′22.3″ E | Olympic Forest Park | Reproductive |
| pot-U3 | Urban | 40°00′18.0″ N | 116°22′53.0″ E | West Beichen Road | Reproductive |
| pot-U4 | Urban | 39°59′08.7″ N | 116°20′03.2″ E | West Zhanchunyuan Road | Reproductive |
| pot-U5 | Urban | 39°52′45.8″ N | 116°25′03.5″ E | Longtan Road | Reproductive |
| sol-U1 | Urban | 39°59′02.1″ N | 116°20′05.4″ E | Beihang University | Vegetative/Reproductive |
| sol-U2 | Urban | 39°52′48.6″ N | 116°24′56.8″ E | Longtan Road | Vegetative/Reproductive |
| sol-U3 | Urban | 39°57′09.6″ N | 116°23′55.1″ E | Qingnianhu Park | Reproductive |
| sol-U4 | Urban | 39°51′43.4″ N | 116°18′21.8″ E | Fengyiqiao Park | Vegetative/Reproductive |
| sol-U5 | Urban | 39°56′01.8″ N | 116°28′38.0″ E | South Road of Beijing Sun Park | Reproductive |
| cir-U1 | Urban | 40°00′28.0″ N | 116°20′14.0″ E | RCEES, Chinese Academy of Sciences | Vegetative |
| cir-U2 | Urban | 40°00′45.8″ N | 116°22′22.3″ E | Olympic Forest Park | Vegetative |
| cir-U3 | Urban | 39°52′48.6″ N | 116°24′56.8″ E | Longtan Road | Vegetative |
| cir-U4 | Urban | 39°57′09.6″ N | 116°23′55.1″ E | Qingnianhu Park | Vegetative |
| cir-U5 | Urban | 39°54′29.3″ N | 116°16′10.1″ E | South Yuyuantan Road | Vegetative |
| pla-U1 | Urban | 40°00′28.0″ N | 116°20′14.0″ E | RCEES, Chinese Academy of Sciences | Reproductive |
| pla-U2 | Urban | 39°58′27.0″ N | 116°24′45.0″ E | East Yinghuayuan Street | Reproductive |
| pla-U3 | Urban | 39°59′08.7″ N | 116°20′03.2″ E | West Zhanchunyuan Road | Reproductive |
| pla-U4 | Urban | 39°52′48.6″ N | 116°25′00.4″ E | Longtan Road | Reproductive |
| pla-U5 | Urban | 39°57′09.6″ N | 116°23′55.1″ E | Qingnianhu Park | Reproductive |
| cre-R1 | Rural | 40°03′50.4″ N | 116°05′52.1″ E | Jiufeng National Forest Park | Reproductive |
| cre-R2 | Rural | 40°03′49.9″ N | 116°05′50.9″ E | Jiufeng National Forest Park | Reproductive |
| cre-R3 | Rural | 40°03′50.1″ N | 116°05′50.0″ E | Jiufeng National Forest Park | Reproductive |
| cre-R4 | Rural | 40°03′46.7″ N | 116°05′48.5″ E | Jiufeng National Forest Park | Reproductive |
| cre-R5 | Rural | 40°03′45.3″ N | 116°05′47.4″ E | Jiufeng National Forest Park | Reproductive |
| set-R1 | Rural | 40°03′50.4″ N | 116°05′52.1″ E | Jiufeng National Forest Park | Reproductive |
| set-R2 | Rural | 40°03′50.1″ N | 116°05′51.8″ E | Jiufeng National Forest Park | Reproductive |
| set-R3 | Rural | 40°03′49.9″ N | 116°05′50.9″ E | Jiufeng National Forest Park | Reproductive |
| set-R4 | Rural | 40°03′51.6″ N | 116°05′52.2″ E | Jiufeng National Forest Park | Reproductive |
| set-R5 | Rural | 40°03′51.4″ N | 116°05′51.9″ E | Jiufeng National Forest Park | Reproductive |
| vio-R1 | Rural | 40°03′50.4″ N | 116°05′52.1″ E | Jiufeng National Forest Park | Reproductive |
| vio-R2 | Rural | 40°03′50.1″ N | 116°05′51.8″ E | Jiufeng National Forest Park | Reproductive |
| vio-R3 | Rural | 40°03′49.9″ N | 116°05′50.9″ E | Jiufeng National Forest Park | Reproductive |
| vio-R4 | Rural | 40°03′50.1″ N | 116°05′50.0″ E | Jiufeng National Forest Park | Reproductive |
| vio-R5 | Rural | 40°03′46.7″ N | 116°05′48.5″ E | Jiufeng National Forest Park | Reproductive |
| inu-R1 | Rural | 40°03′50.1″ N | 116°05′51.8″ E | Jiufeng National Forest Park | Vegetative |
| inu-R2 | Rural | 40°03′49.9″ N | 116°05′50.9″ E | Jiufeng National Forest Park | Vegetative |
| inu-R3 | Rural | 40°03′50.1″ N | 116°05′50.0″ E | Jiufeng National Forest Park | Vegetative |
| inu-R4 | Rural | 40°03′46.7″ N | 116°05′48.5″ E | Jiufeng National Forest Park | Vegetative |
| inu-R5 | Rural | 40°03′51.6″ N | 116°05′52.2″ E | Jiufeng National Forest Park | Vegetative |
| pot-R1 | Rural | 40°03′50.4″ N | 116°05′52.1″ E | Jiufeng National Forest Park | Reproductive |
| pot-R2 | Rural | 40°03′51.6″ N | 116°05′52.2″ E | Jiufeng National Forest Park | Reproductive |
| pot-R3 | Rural | 40°03′50.9″ N | 116°05′51.6″ E | Jiufeng National Forest Park | Reproductive |
| pot-R4 | Rural | 40°03′50.2″ N | 116°05′51.0″ E | Jiufeng National Forest Park | Reproductive |
| pot-R5 | Rural | 40°03′53.6″ N | 116°05′50.4″ E | Jiufeng National Forest Park | Reproductive |
| sol-R1 | Rural | 40°03′50.1″ N | 116°05′51.8″ E | Jiufeng National Forest Park | Vegetative |
| sol-R2 | Rural | 40°03′50.1″ N | 116°05′50.0″ E | Jiufeng National Forest Park | Vegetative |
| sol-R3 | Rural | 40°03′45.3″ N | 116°05′47.4″ E | Jiufeng National Forest Park | Vegetative |
| sol-R4 | Rural | 40°03′51.6″ N | 116°05′52.2″ E | Jiufeng National Forest Park | Vegetative |
| sol-R5 | Rural | 40°03′51.4″ N | 116°05′51.9″ E | Jiufeng National Forest Park | Vegetative |
| cir-R1 | Rural | 40°03′51.6″ N | 116°05′52.2″ E | Jiufeng National Forest Park | Vegetative |
| cir-R2 | Rural | 40°03′51.4″ N | 116°05′51.9″ E | Jiufeng National Forest Park | Vegetative |
| cir-R3 | Rural | 40°03′53.2″ N | 116°05′52.3″ E | Jiufeng National Forest Park | Vegetative |
| cir-R4 | Rural | 40°03′53.3″ N | 116°05′51.8″ E | Jiufeng National Forest Park | Vegetative |
| cir-R5 | Rural | 40°03′53.6″ N | 116°05′50.4″ E | Jiufeng National Forest Park | Vegetative |
| pla-R1 | Rural | 40°03′50.4″ N | 116°05′52.1″ E | Jiufeng National Forest Park | Reproductive |
| pla-R2 | Rural | 40°03′49.9″ N | 116°05′50.9″ E | Jiufeng National Forest Park | Reproductive |
| pla-R3 | Rural | 40°03′46.7″ N | 116°05′48.5″ E | Jiufeng National Forest Park | Reproductive |
| pla-R4 | Rural | 40°03′45.3″ N | 116°05′47.4″ E | Jiufeng National Forest Park | Reproductive |
| pla-R5 | Rural | 40°03′51.6″ N | 116°05′52.2″ E | Jiufeng National Forest Park | Reproductive |

RCEES, Research Center for Eco-Environmental Science. *Cre*, *Crepidiastrum sonchifolium*; *Set*, *Setatia viridis*; *Vio*, *Viola philippica*; *Inu*, *Inula japonica*; *Pot*, *Potentilla supina*; *Sol*, *Solanum nigrum*; *Cir*, *Cirsium setosum*; *Pla*, *Plantago depressa*.

**Supplemental Table 2** Linear regression of the plant rank of nestedness to sampling spatial distance, environmental distance, and fungal variation in urban and rural areas.

|  | Urban | |  | Rural | |
| --- | --- | --- | --- | --- | --- |
|  | Slope | *P* value |  | Slope | *P* value |
| Spatial distance | 0.124 | 0.905 |  | -0.557 | 0.134 |
| Environmental distance | 0.292 | 0.482 |  | -0.161 | 0.871 |
| Fungal variation | **0.803** | **0.016** |  | 0.005 | 0.991 |

Use data of 8 host species (n=8). The mean fungal Bray-Curtis distance among replications of the same host species is used as an indicator of fungal variation. Mean spatial distance among replications of the same host species is used as an indicator of spatial distance. The mean soil environmental Euclidean distance among replications of the same host species is used as an indicator of environmental distance.

**Supplemental Table 3 Description of sequences corresponding to the closest matches from the reference database of the representative sequence of each OTU in *Glomus* Group Ⅱ.**

| OTU ID | Organism | Accession | Sample source | Habitat description | Reference |
| --- | --- | --- | --- | --- | --- |
| OTU 12 | uncultured *Glomus* | AB698612 | Roots of grass | Steppe zone of Mongolia, with various degrees of degradation due to grazing | Goomaral et al. (2013) |
| OTU 20 | uncultured *Glomus* | AB749496 | Roots of grass | Steppe zone of Mongolia, with various degrees of degradation due to grazing | Goomaral et al. (2013) |
| OTU 40 | uncultured *Glomus* | AB698612 | Roots of grass | Steppe zone of Mongolia, with various degrees of degradation due to grazing | Goomaral et al. (2013) |
| OTU 42 | *Glomus* sp. Glo38 | AY129612 | Plant roots | Republic of Panama, Tropical forest | Husband et al. (2002) |
| OTU 50 | uncultured *Glomus* | GU183691 | Plant roots/ soils | Loess Plateau region of northwest China, a long-term fertilization experiment site | Wu et al. (2011) |
| OTU 74 | uncultured *Glomus* | AB749496 | Roots of grass | Steppe zone of Mongolia, with various degrees of degradation due to grazing | Goomaral et al. (2013) |

**Reference cited in Tables**

Goomaral, A., Iwase, K., Undarmaa, J., Matsumoto, T., Yamato, M. (2013). Communities of arbuscular mycorrhizal fungi in *Stipa krylovii* (Poaceae) in the Mongolian steppe. *Mycoscience* 54, 122-129. doi: 10.1016/j.myc.2012.09.006

Husband, R., Herre, E.A., Turner, S.L., Gallery, R., Young, J.P.W. (2002). Molecular diversity of arbuscular mycorrhizal fungi and patterns of host association over time and space in a tropical forest. *Mol Ecol* 11, 2669-2678. doi: DOI 10.1046/j.1365-294X.2002.01647.x

Wu, F.S., Dong, M.X., Liu, Y.J., Ma, X.J., An, L.Z., Young, J.P.W., et al. (2011). Effects of long-term fertilization on AM fungal community structure and Glomalin-related soil protein in the Loess Plateau of China. *Plant Soil* 342, 233-247. doi: 10.1007/s11104-010-0688-4
